# Derivation of inter-lamellar behaviour of the intervertebral disc annulus

**DOI:** 10.1016/j.jmbbm.2015.03.028

**Published:** 2015-08

**Authors:** Marlène Mengoni, Bethany J. Luxmoore, Vithanage N. Wijayathunga, Alison C. Jones, Neil D. Broom, Ruth K. Wilcox

**Affiliations:** a Institute of Medical and Biological Engineering, School of Mechanical Engineering, University of Leeds, Leeds LS2 9JT, UK; b University of Leeds, UK; c University of Auckland, NZ

**Keywords:** Inter-lamellar behaviour, Annulus fibrosus, Optimisation, Cohesive interface, Calibration

## Abstract

The inter-lamellar connectivity of the annulus fibrosus in the intervertebral disc has been shown to affect the prediction of the overall disc behaviour in computational models. Using a combined experimental and computational approach, the inter-lamellar mechanical behaviour of the disc annulus was investigated under conditions of radial loading.

Twenty-seven specimens of anterior annulus fibrosus were dissected from 12 discs taken from four frozen ovine thoracolumbar spines. Specimens were grouped depending on their radial provenance within the annulus fibrosus. Standard tensile tests were performed. In addition, micro-tensile tests under microscopy were used to observe the displacement of the lamellae and inter-lamellar connections. Finite elements models matching the experimental protocols were developed with specimen-specific geometries and boundary conditions assuming a known lamellar behaviour. An optimisation process was used to derive the interface stiffness values for each group. The assumption of a linear cohesive interface was used to model the behaviour of the inter-lamellar connectivity.

The interface stiffness values derived from the optimisation process were consistently higher than the corresponding lamellar values. The interface stiffness values of the outer annulus were from 43% to 75% higher than those of the inner annulus. Tangential stiffness values for the interface were from 6% to 39% higher than normal stiffness values within each group and similar to values reported by other investigators. These results reflect the intricate fibrous nature of the inter-lamellar connectivity and provide values for the representation of the inter-lamellar behaviour at a continuum level.

## 1 Introduction

The annulus fibrosus is the fibrous avascular outer region of the intervertebral disc. It comprises a series of almost circumferential layers termed lamellae (Cassidy et al., 1989). Each lamella is composed of collagen fibre bundles embedded within a ground matrix. The fibres can be mechanically characterised as non-linear entities acting in tension only (Skaggs et al., 1994; Holzapfel et al., 2005). Secondary structures such as bridges across the lamellae and a surrounding elastin network are also present (Yu et al., 2002; Pezowicz et al., 2006; Schollum et al., 2008).

A realistic virtual model of the intervertebral disc can give insight into the significance of deformation patterns in its naturally loaded configuration as well as post-trauma or post-clinical procedures. It can be a tool for the design of novel treatment approaches for the disc, anticipating outcomes of clinical procedures, including related tissue damage, and accounting for population variations. An understanding of the mechanical properties of the different constituent tissues, their interactions, and their role in the functional mechanics of the intervertebral disc is of major importance in the development of such models. In the last decade, many constitutive models of the annulus fibrosus have been proposed, most considering the solid phase of the annulus to be composed of two sets of fibres with oblique-counter oblique orientations and an isotropic elastic matrix (Elliott and Setton, 2000; Eberlein et al., 2001; Noailly et al., 2011; Galbusera et al., 2011; Cortes et al., 2013; Marini and Ferguson, 2014), with a number also accounting for some of the secondary structures between adjacent lamellae (Peng et al., 2006; Caner et al., 2007; Guerin and Elliott, 2006; Hollingsworth and Wagner, 2011; Reutlinger et al., 2014; Labus et al., 2014). The nature of inter-lamellar connectivity has only recently been studied, focussing mainly on the additional shear strength that it provides to the overall structure (Iatridis and Gwynn, 2004; Gregory et al., 2011, 2014; Kirking et al., 2014; Labus et al., 2014). It has been shown that accounting for the inter-lamellar connectivity in the annulus fibrosus predicts significantly different strain and stress distributions in the intervertebral disc under physiological loading (Hollingsworth and Wagner, 2011; Luxmoore, 2013; Mengoni et al., 2013; Reutlinger et al., 2014). When the overall behaviour of the annulus is of interest and not its localised patterns of deformation, the inter-lamellar connectivity can be omitted, provided the material parameters are calibrated against mechanical data acquired on samples including several lamellae (such as in Guerin and Elliott, 2006; Jacobs et al., 2011). However, the assumed lamellar behaviour is often calibrated against single lamella mechanical tests (Eberlein et al., 2001, 2004; Noailly et al., 2011; Galbusera et al., 2011; Malandrino et al., 2013; Marini and Ferguson, 2014; Reutlinger et al., 2014). The inclusion of the inter-lamellar connectivity, explicitly or implicitly, in those in-silico models is then necessary.

The aim of the present study was to develop an explicit representation of inter-lamellar connectivity within a finite element model of the annulus fibrosus. The focus was on the linear elastic inter-lamellar behaviour rather than viscosity, damage, or other non-linear effects. To this end, a parallel experimental and computational study was used to derive the interface stiffness under radial loading of the disc annulus.

## 2 Material and methods

A combined experimental and computational approach was developed to calibrate the linear part of an interface model of the inter-lamellar mechanical behaviour of the annulus fibrosus.

### 2.1 Specimen preparation and tensile testing

**Fig. 1:**
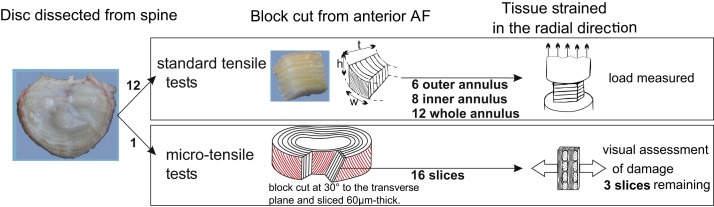
Experimental workflow. Top frame: standard tensile tests showing a sketch of a typical specimen with references to the directions and dimensions: *t* radial direction, specimen thickness; *h*: axial direction, specimen height, and *w*: circumferential direction, specimen width; Bottom frame: micro-tensile tests showing orientation of the specimen blocks.

The ovine disc was chosen for this study as its biochemical and structural properties are qualitatively similar to those of the human disc (Wilke et al., 1997; Alini et al., 2008); it is also commonly used as an in-vivo model (Reitmaier et al., 2012; Hegewald et al., 2015) and therefore presents an interest in its own right. Ovine and human discs however have somewhat different morphologies and no direct quantitative comparisons is offered at the full disc level. Mature ovine spines obtained from an abattoir were used in this study. All discs were extracted from the frozen thoracolumbar spines stored at −20 °C in a plastic wrap and were classified as having a low level of degeneration by two independent reviewers (grade I or II following Thompson et al., 1990 scale). Anterior annulus intervertebral disc samples were carefully extracted from all levels in the thoracolumbar spine. Two types of protocols were developed: one for micro-tensile testing and the other as a standard tensile test (see Fig. 1).

**Fig. 2:**
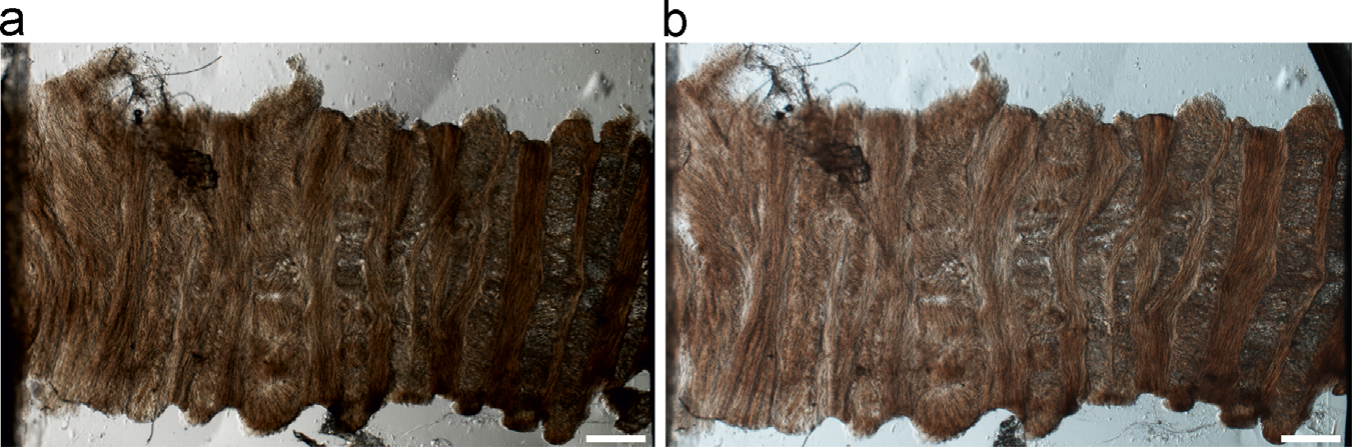
(a) Microscopy image of a radial annulus slice. (b) Same slice stretched by a total of 0.5 mm. The white bars are 200 μm scales.

For micro-tensile testing, one T13-L1 annulus sample was mounted in a freezing stage sledge microtome and sectioned (60 μm thickness) at 30° to the transverse plane. This enabled the extraction of sections where alternate lamellae were cut in alignment with their fibres, as described in Pezowicz et al. (2006) and Schollum et al. (2009). The sections (*N*=16) were floated in saline and tested within two hours of extraction from the disc. They were individually placed into a bespoke tensile testing rig (Broom, 1986 and Pezowicz et al., 2006) with the radial ends of the section securely clamped. The rig was mounted directly into the rotating stage of a conventional light microscope (Multizoom AZ100, Nikon Instruments). The fully hydrated annulus sections were strained in tension by 0.5 mm in the radial direction; no load measurement was performed. Images of the initial state and strained sections were captured (Fig. 2). Sections that had been damaged during dissection, cryosectioning, or loading were eliminated from the study. The images from the acceptable sections, at rest and strained, were analysed to extract the region that was visible on each image pair. The sections with less than six lamellae in this region were discarded from further analysis. For the remaining tested sections (*N*=3) an algorithm was written (ScanIP 5.1, Simpleware Ltd., UK and ImageJ, U.S. N.I.H., USA) to identify the interfaces between lamellae and the external edges of the tissue. In order to track displacement of the tissue, 10 characteristic points at the lamellar interfaces were used, along with the external edges of the lamellae in the visible region.

For the standard tensile tests, annulus samples (*N*=26) extracted from a total of 11 discs of three lumbar spines were prepared as rectangular parallelepiped specimens (see Fig. 1). They were divided into three groups depending on their radial location in the anterior annulus: outer annulus (*N*=6), inner annulus (*N*=8) and samples across the whole annulus thickness (*N*=12). The dimensions of each specimen were recorded as well as the number of lamellae per sample and the fibre direction in the outer-most layer. The outer-most layer was glued onto the end of an axial cylinder fitting into a materials testing machine (Instron 5543, Instron Corp., Norwood, MA, fitted with a 50N static load cell). The inner-most layer of the specimen was glued to the bottom plate of the testing machine in such a way that the specimen was compressed by less than 1%, as measured by the distance between the testing plates. This initial compression was produced to achieve full contact while the adhesive was setting and a preliminary study showed it was low enough not to create any damage. Tensile radial tests were performed at a crosshead speed of 1 mm/min while recording the load. Each specimen was extended to a tensile strain of at least 20%. All specimens were floated in 0.10 M saline solution after preparation as parallelepiped samples and prior to tensile testing, and tested within an hour of the disc extraction from the frozen spine. The dimensions of a subset of 5 samples were measured before and after the saline bath. No change in dimensions was observed within the measurement precision. Each tensile test lasted less than 5 min. The radial modulus was derived for each sample as the slope of a linear fit between 0.5% and 20% tensile strain. The first 0.5% tensile strain was discarded to suppress boundary contact effects. The measured radial modulus was compared between groups. Given the low number of samples and the non-Gaussian nature of the data, the specimen moduli were compared using a Kruskal–Wallis test and the corresponding paired comparisons with Mann–Whitney tests using statistical software (R.3.1.1, R foundation for statistical computing). Statistical significance was set at *p*<0.01.

### 2.2 Finite element modelling

Two computational approaches were developed to match the experimental protocols: 2D section-specific models of the micro-tensile tests and 3D generic models of the standard tensile tests. Each model was developed with the geometry and boundary conditions based on the experimental configurations.

**Fig. 3:**
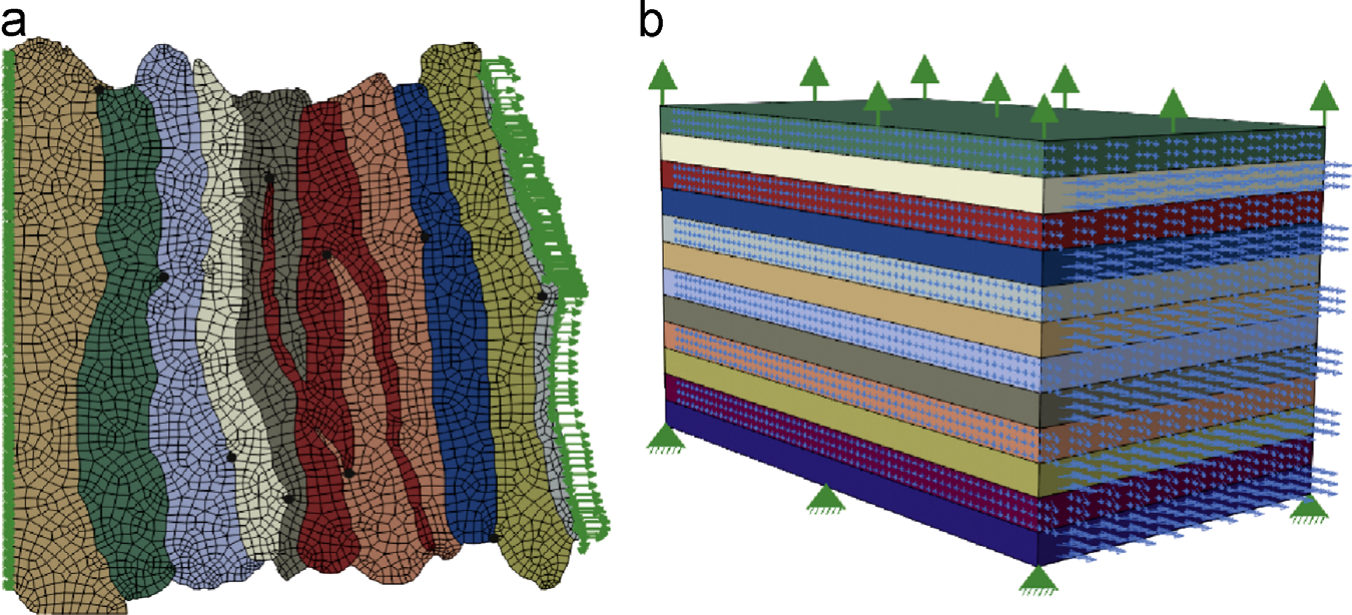
(a) Finite element quadrilateral mesh and boundary conditions of the slice pictured in Fig. 2, the black dots represent the position of the points at which displacement is extracted; (b) Generic geometry and boundary conditions for the standard tensile tests models. The blue arrows represent the fibre direction in each lamella (For interpretation of the references to colour in this figure legend, the reader is referred to the web version of this article.).

For the micro-tensile tests, 2D section-specific geometries were acquired from the edges and interfaces extracted from the microscopy images. 4-node quadrilateral meshes were built, using Abaqus 6.13 (Simulia, Dassault Système), preserving the external and interface edges of each lamella. The Python code, 2DImage2Mesh, associated with this procedure is available from Github (Mengoni, 2015a). The measured external displacement of each section was applied as boundary conditions to each 2D plane-stress model (Fig. 3a).

For the standard tensile tests, the specimens were modelled as rectangular parallelepipeds, each with the specimen-specific dimensions and number of lamellae. The lamellar thickness was assumed constant and dependent on the specimen thickness. The 3D models were meshed with 8-node hexahedral elements with selective reduced integration. The experimental tensile tests were replicated assuming perfect adhesion of the specimen to the testing machine. Zero displacement was imposed on one side, while the nodes of the opposite side were translated in the radial disc direction by 20% of the specimen thickness (Fig. 3b).

**Fig. 4:**
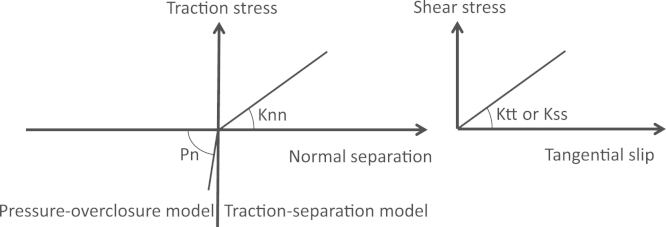
Stress–displacement behaviour of the interface model. The interface stiffness is given by the cohesive stiffness values (*K*_nn_/*K*_tt_/*K*_ss_) when the separation between two surfaces is positive while it is proportional to the contact penalty (*P*_n_) when the separation is negative.

In both cases, each lamella interacted mechanically with its neighbours through the simulation of a surface-based inter-lamellar cohesive behaviour (Fig. 4). The interface behaviour was described either by a pressure-overclosure law when two surfaces penetrate each other or by a linear traction-separation law when two surfaces separate. The traction stress of the pressure-overclosure model was proportional to the negative separation between the surfaces, the proportionality coefficient being the normal penalty. The traction-separation law was similarly described by normal and shear stresses, assuming those vary linearly with the surface separation. The normal stress was proportional to the normal separation through the normal cohesive stiffness *K*_nn_. The shear stresses were proportional to the tangential slips through the tangential cohesive stiffness values *K*_tt_ and *K*_ss_. This surface-based cohesive model is described as decoupled because the normal separation does not influence the shear stresses in the traction-separation law and the tangential slip does not influence the normal stress.

A hyperelastic Holzapfel model (Eberlein et al., 2001; Gasser et al., 2006) with one fibre direction was used for the lamellar material. The annulus was assumed to be composed of an isotropic ground matrix (Neo-Hookean model) embedded with collagen fibres bearing load in tension only:

(1) $$W=\frac{1}{2}K\;{\left(J-1\right)}^{2}+\frac{1}{2}G\;\left(I_{1}-3\right)+\;\frac{k_{1}}{2k_{2}}(e^{k_{2}{\left(I_{4,a}-1\right)}^{2}}-1)$$

where *W* is the strain energy density function; *K*, *G* are respectively the bulk modulus of the annulus, the shear modulus of the annulus ground matrix. $k_{1}$ and $k_{2}$ are fibre related parameters describing the exponential stress–strain behaviour. $I_{1}$, $I_{4,a}$ and J are invariants of the Right Cauchy-Green tensor *C*:

(2) $$I_{1}=\;tr(C)$$

(3) $$I_{4,a\;}=a^{T}:C:a$$

(4) $$J^{2}=\det(C)$$

where a is the unit vector representing the collagen fibre orientation in the reference configuration, expressed with respect to the global coordinate system of the model.

**Fig. 5:**
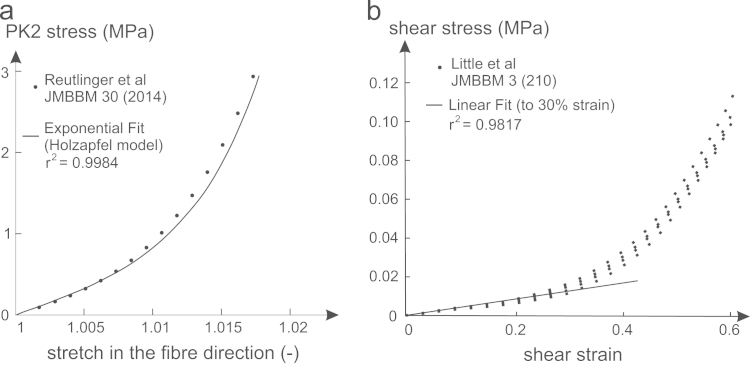
(a) Exponential fit to the model from Reutlinger et al. (2014) used to calibrate $k_{1}$ and $k_{2}$, and (b) linear fit to the shear data from Little et al. (2010) used to calibrate *G*.

Material parameters $k_{1}$ and $k_{2}$ were derived from Reutlinger et al. (2014), see Table 1 and Fig. 5(a). While the fibre contribution in the constitutive model used in Reutlinger et al. (2014) at the lamellar level was modelled from another strain energy density (Markert et al., 2005), the one-dimensional aspect of the fibres allowed a direct analytical comparison between models. The parameters $k_{1}$ and $k_{2}$ were adjusted to fit the data for fibre extensions up to 2% (i.e. the entire range of extension in Reutlinger et al., 2014). The fibre contribution data from the study of Reutlinger et al. (2014) was chosen as a baseline for the material parameters as it was one of the few studies to include material parameters specific to the anterior annulus of ovine intervertebral discs. The shear modulus *G* was calibrated against experimental shear data on the ground matrix embedded with fibres (Little et al., 2010) with a linear regression from 0 to 30% shear strain, see Table 1 and Fig. 5(b). The bulk modulus *K* was approximated as the bulk modulus of water, i.e. a nearly incompressible material (see Table 1).

For the micro-tensile test models, the 2D nature of the finite elements required the fibre orientation to be within the analysis plane. The alternate angle between lamellae meant that alternate lamellae contained fibres with an out-of-plane orientation (see Fig. 1). This was accounted for in the 2D model by projecting the fibre orientation onto the plane for the lamellae that were cut with cross-sectioned fibres. For the standard tensile tests, the fibre angle of the outer-most layer was matched to measurements from the experimental sample and this was also used for alternate layers throughout the sample, while those in between were given a supplementary angle orientation.

All finite element analyses were non-linear quasi-static implicit and run in parallel with Abaqus 6.13 (Simulia, Dassault Système).

### 2.3 Optimisation process

A quasi-Newton optimisation process using the L-BFGS-B algorithm (Byrd et al., 1995; Zhu et al., 1997) was used to derive the cohesive stiffness values *K*_nn_/*K*_tt_/*K*_ss_. The cost function was the least-square normalised difference between experimental data and the corresponding finite element results. The L-BFGS-B implementation in SciPy (Python 2.7, www.python.org) was used in this work.

For the micro-tensile tests, the displacement was extracted at 10 points per specimen, matching the experimental measurement points. The data used for the cost function were the displacements in each direction for each of the 10 points, i.e. 20 values per model. The optimisation process was completed separately on the three modelled slices, yielding one set of interface stiffness values per slice.

For the standard tensile tests, the data used for the cost function was the specimen-specific radial stiffness values, i.e. eight values for the outer annulus group, six values for the inner annulus group, and 12 values for the whole thickness annulus group. The optimisation process was completed for each group of specimens, yielding one set of interface stiffness values per group.

Initial interface stiffness values were chosen to match the lamellar radial stiffness. This lamellar radial stiffness, $k_{\mathrm{matrix}}$, is the shear modulus assumed for the ground matrix, *G*, converted to a Young׳s modulus assuming a linear behaviour, and normalised against the mean lamellar thickness, *t*:

(5) $$k_{\mathrm{matrix}}=\frac{\left(1+\nu \right)\;2G}{t}$$

For the micro-tensile tests, this initial parameter values have been varied to identify possible local minima of the optimisation process.

**Fig. 6:**
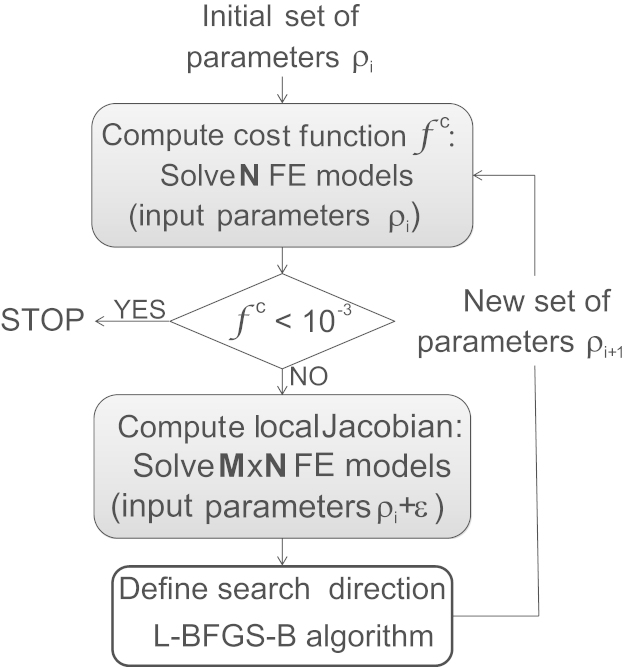
Optimisation process. In the present case, the parameters $\rho_{i}$ are the interface stiffness values, ***N*** is 1 for the micro-tensile tests and the number of samples per group for the standard tensile tests, ***M*** is the number of parameters, i.e. 2 or 3, and *ε* is an arbitrary small correction applied to each parameter in order to establish the search direction. The L-BFGS-B algorithm may involve computing the cost function several other times.

At each iteration of the optimisation algorithm, the finite element problems were solved to compute the value of the cost function as well as the local Jacobian matrix of the optimisation problem (Fig. 6). The optimisation process was terminated when the cost function achieved a value of 10^−3^. The Python code, opti4Abq, associated with this procedure is available from Github (Mengoni, 2015b).

## 3 Results

The data associated with this paper (microscopy images, geometrical dimensions, model input files and results) are openly available from the University of Leeds Data Repository (Mengoni and Wilcox, 2015).

### 3.1 Experimental mechanical tests

An example of sample subjected to extension with the micro-tensile test protocol is presented in Fig. 2. It shows that the lamellae sustain most of the deformation in the samples, with a low level of localised delamination for radial extensions of 0.5 mm, i.e. 12–23% strain. Similar observations were made for all samples.

**Fig. 7:**
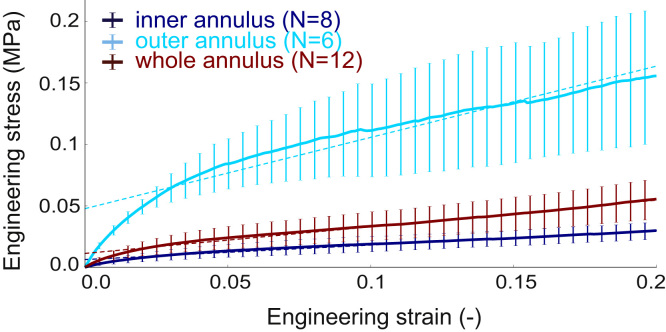
Stress–strain experimental data (means±standard deviations) of the standard tensile tests for each group. Plain line: experimental data, dashed lines: linear fit.

For the standard tensile tests, cross section area (disc height×circumferential width), specimen thickness, and measured experimental moduli are reported in Table 2. The engineering stress vs. engineering strain data are plotted in Fig. 7. The applied forces ranged from 0 to 4.5 N, with a precision error due to the tensile testing machine of 0.25%. The modulus of the outer annulus group was significantly higher than the modulus of the inner annulus (*p*=6.66×10^−4^) and whole annulus thickness (*p*=3.23×10^−3^) groups. The linear fit used to calculate the modulus from the experimental curves showed a good approximation for the range of strains used (least square residuals below 6% for each specimen).

### 3.2 Optimisation process and interface stiffness

For all micro-tensile models, the optimisation process converged with normal termination in eight iterations. The displacement magnitude error was below 10% of the measured displacement at all points. The standard tensile test models converged with normal termination in 9, 15, and 13 iterations, solving a total of 1096, 1782, and 2112 finite element models respectively for the outer annulus, inner annulus and whole annulus thickness groups. The specimen stiffness error was below 5% for each specimen. Varying the initial stiffness values yielded no difference in the final results of the optimisation process for values above 15%, the baseline value given by $k_{matrix}$.

**Fig. 8:**
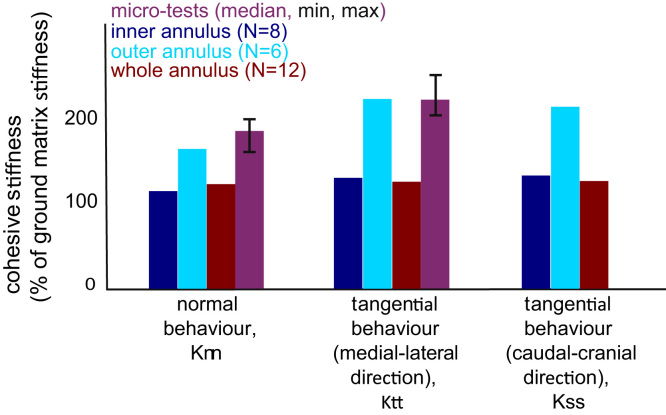
Cohesive interface stiffness values of the micro-tensile tests models (median, minimal and maximal values) and standard tensile tests models. Values are depicted as percentage values of the tissue matrix stiffness. The tangential stiffness of the micro-tensile tests is in the direction parrallel to one of the fibre orientations, i.e. in the plane of analysis.

The computed inter-lamellar stiffness values for each sample (micro-tensile test models) or each group (standard tensile test models) are plotted in Fig. 8 with respect to the corresponding lamellar values i.e. lamellar surface stress normalised by lamellar extension. The interface stiffness values were consistently higher than the lamellar stiffness values. In the standard tensile tests models, the interface stiffness values of the inner annulus samples were always smaller than those of the whole annulus thickness group. The interface stiffness values of the outer annulus were from 43% to 75% higher than those of the other groups. Finally, the tangential stiffness values were from 6% to 39% higher than the normal stiffness value.

## 4 Discussion

### 4.1 Significance

The connectivity between the lamellae of the annulus fibrosis affects the overall mechanical performance of the tissue but this inter-lamellar behaviour has yet to be fully represented in computational models of the intervertebral disc. In this study, the linear part of the inter-lamellar behaviour of the annulus fibrosus was investigated using a combined experimental and computational approach, assuming a known lamellar behaviour.

In terms of the overall behaviour of the tissue from the experimental results, given that the loading direction was always perpendicular to the lamellar fibre direction, the experimental modulus in the direction of loading was expected to be similar to the assumed matrix modulus. This was observed for the inner annulus specimens as well as for the specimens extending over the whole annulus thickness, where the load–displacement behaviour was dominated by the inner annulus tissue. The outer annulus however had a significantly higher modulus. This is in agreement with previous studies that have shown radial variations in the mechanical behaviour of the annulus fibrosus (Fujita et al., 2000; Holzapfel et al., 2005). In term of a quantitative comparison, Fujita et al. (1997) reported radial tangent modulus values for the inner and middle annulus in the human tissue that are three times higher than the one presented in the current work. This difference can be explained by the different species from which the tissue was taken, human or ovine. Indeed Schmidt and Reitmaier (2013) reported from an in-silico study that the aggregate modulus of ovine annulus tissue was about nine times lower than that of human tissue.

Importantly, this study has shown that the computed inter-lamellar stiffness values are consistently higher than the corresponding lamellar values. This reflects the intricate fibrous nature of the inter-lamellar connections. Connections between lamellae are made via a network of elastin fibres and collagen cross-bridges (Yu et al., 2002). In particular, as shown by Pezowicz et al. (2006), radial bridging structures between near-neighbour lamellae of the same fibre orientation are present across the whole anterior annulus. Those bridging structures are discrete entities of continuous fibrous nature and are highly integrated with the collagen fibres of the lamellae (Schollum et al., 2008). This collagen-based connection is likely to limit the slip between adjacent lamellae and thus contribute to the inter-lamellar stiffness values reported in the present study.

The inter-lamellar stiffness values obtained from the micro-tensile tests were calibrated against overall deformation of the samples while for the standard tensile tests an apparent radial stiffness was the calibration target. However, the ratio between lamellar and inter-lamellar radial stiffness values are similar for both types of tests, suggesting the set of stiffness values yielded by the optimisation process are global values and not local minima of the optimisation function. The latter are however reached when the initial guess for the inter-lamellar stiffness values is a large underestimation of the actual value. In this case, the cohesive behaviour yields a very large separation for a low force, and minimising the displacement difference is not possible.

Even though only radial tensile tests were performed, the tangential stiffness values obtained for both the inner annulus and whole annulus thickness samples are of the same order as other reported values (Gregory et al., 2011; Labus et al., 2014) obtained using lap tests or shear experiments. This confirms the validity of the method employed in the present study to characterise the complete inter-lamellar behaviour and not just the radial interface stiffness. The difference seen in the inter-lamellar stiffness values between the medial–lateral and the caudal–cranial directions can be explained by the presence of the fibres in the model. Their contribution in each of those directions is different because their orientation is not symmetrical with respect to those directions.

### 4.2 Limitations and challenges

The results are however subject to some limitations. The micro-tensile tests models were assessed by measuring the displacement of 10 characteristic points in the observed stretched slices. The use of advanced image analyses techniques such as digital image correlation (Guerin and Elliott, 2006; Michalek et al., 2009) would probably have given more accurate results as it would have provided a more continuous displacement field. However, the method used in this study allowed for an efficient use of an optimisation algorithm with a cost function evaluated on 20 values that resulted in low local errors. Moreover, the displacement field between each characteristic point was smooth enough to be matched with a low spatial discretisation. An advanced image correlation technique would be needed for larger strain models that would likely yield larger variations in the displacement field.

Only the interface stiffness was varied between groups in the standard tensile tests models. However the difference in specimen modulus between outer and inner annulus may be due to a combination of different effects. In particular, the lamellar behaviour was considered homogeneous, and derived from the literature data, through the whole annulus thickness. While the loading mode involved fibre stretch only through an overall Poisson effect and the fibre behaviour is thus less likely to influence the results, it is possible that the matrix modulus might contribute to the measured differences in the specimen modulus. Similarly, the lamellar thickness was assumed constant over a specimen while in reality the outer lamellae were observed to be thicker than the inner ones. In addition, pre-stress was not applied in this study because it was considered likely to be minimal due to the orientation of the specimens perpendicular to the principal fibre directions. In this study, the lamellar parameters themselves were not calibrated against the experimental data used for the inter-lamellar behaviour because of the relatively low number of experimental data.

The collagen bridging structures and other inter-lamellar connections contributing to the inter-lamellar stiffness are discrete, localised entities (Schollum et al., 2008) while the computational approach used here is a continuous representation of the interface between lamellae. This continuous interface is therefore not representative of the local sub-lamellar behaviour of the tissue. It is, however, representative of a homogenised approach at the lamellar level. As a specimen-specific sub-lamellar architecture is not available clinically, the continuous approach at the lamellar level is not limiting the prospective clinical applications of the model. Moreover the interface behaviour assumed non-coupled normal and tangential bonding forces to reduce the number of unknowns to three. The fibrous aspect of the inter-lamellar connectivity could contribute to coupling these forces, the tangential forces being influenced by the normal separation and the normal forces by the tangential slips. However, given the relatively small amount of experimental data on which the model was calibrated, it was more preferable to reduce the number of unknowns to three and assume a decoupled behaviour.

The methods used in this study present a number of challenges, related to both the experimental and computational approaches. The development of specimen-specific models of the micro-tensile tests requires intact slices of annulus to be mechanically tested. Keeping annulus slices intact, whether during cryosectioning, positioning on the testing rig, clamping, or during testing, proved to be a very delicate process. While 16 sectioned slices were intact, only 3 loaded slices were considered as undamaged and large enough to be able to capture the displacement during loading.

From a computational point of view, each finite element model developed has a relatively low computational cost. However, the number of models solved in the optimisation process requires careful implementation of parallel computation. For example, in this study the models were solved using a high performance computer with 16 parallel CPU and 128GB RAM. The running wall clock time for the least demanding models was below 5 min whereas the most time-consuming models required approximately 1.5 h. As the optimisation process required between 1000 and 2000 models to run, the total time for the termination of the optimisation process, running 4 models in parallel was about 8 days.

### 4.3 Conclusions

The new results reported in this study can be used to produce an annulus fibrosus finite element model that explicitly describes the inter-lamellar behaviour. The interface stiffness is likely to play a greater role than the lamellae ground matrix stiffness in the overall radial behaviour of the annulus fibrosus. Description of the inter-lamellar behaviour is needed for models in which the lamellar behaviour is calibrated against single lamella mechanical data. A model explicitly including the lamellae will be useful for predicting the inter-lamellar deformations in micro-mechanical models of the intervertebral disc. In particular, when further combined with damage and failure models to describe the non-linear part of the inter-lamellar connectivity, such a model can contribute to the prediction of clinically induced damage as would, for example, be caused by a percutaneous procedure. The explicit inclusion of interface behaviour will permit modelling of the delamination caused by local disruption such as perforations due to trauma or surgical procedure and the presence of repair devices.

## Figures and Tables

**Table 1:** Material parameters of the Holzapfel model (derived from Little et al., 2010 and Reutlinger et al., 2014).

*K* (MPa)	*G* (MPa)	$k_{1}$ (MPa)	$k_{2}$ (−)
2200	0.06	23.92	1045.7

**Table 2:** Dimensions and measured modulus of the standard tensile test sample groups.

	Cross section area (mm^2^)		Specimen thickness (mm)		Specimen modulus (MPa)		Strain rate (%/min)	
	Mean	Std	Mean	Std	Mean	Std	Mean	Std
Outer annulus (*N*=8)	26.46	10.65	3.42	0.81	0.580	0.279	30.8	7.0
Inner annulus (*N*=6)	23.81	8.38	3.59	0.44	0.118	0.035	28.3	3.7
Whole annulus (*N*=12)	20.50	3.39	4.52	0.87	0.216	0.076	23.0	4.8
